# Endocytosis in the adaptation to cellular stress

**DOI:** 10.15698/cst2020.10.232

**Published:** 2020-08-18

**Authors:** Tania López-Hernández, Volker Haucke, Tanja Maritzen

**Affiliations:** 1 Leibniz-Forschungsinstitut für Molekulare Pharmakologie (FMP), 13125 Berlin, Germany.; 2 Freie Universität Berlin, Faculty of Biology, Chemistry, Pharmacy, 14195 Berlin, Germany.

**Keywords:** clathrin-mediated endocytosis, cancer, nutrient signaling, osmotic stress, mechanical stress, hypoxia, oxidative stress

## Abstract

Cellular life is challenged by a multitude of stress conditions, triggered for example by alterations in osmolarity, oxygen or nutrient supply. Hence, cells have developed sophisticated stress responses to cope with these challenges. Some of these stress programs such as the heat shock response are understood in great detail, while other aspects remain largely elusive including potential stress-dependent adaptations of the plasma membrane proteome. The plasma membrane is not only the first point of encounter for many types of environmental stress, but given the diversity of receptor proteins and their associated molecules also represents the site at which many cellular signal cascades originate. Since these signaling pathways affect virtually all aspects of cellular life, changes in the plasma membrane proteome appear ideally suited to contribute to the cellular adaptation to stress. The most rapid means to alter the cell surface proteome in response to stress is by alterations in endocytosis. Changes in the overall endocytic flux or in the endocytic regulation of select proteins conceivably can help to counteract adverse environmental conditions. In this review we summarize recent data regarding stress-induced changes in endocytosis and discuss how these changes might contribute to the cellular adaptation to stress in different systems. Future studies will be needed to uncover the underlying mechanisms in detail and to arrive at a coherent picture.

## INTRODUCTION

Cells have to cope with challenging changes in their environment such as alterations in osmolarity, ion homeostasis, redox state, oxygen or nutrient supply, or exposure to ionizing radiation or potentially toxic substances. Deviations from homeostatic conditions are often unfavorable to cellular life and elicit stress responses that have evolved to foster adaptation to the altered conditions and, thereby, promote cellular survival in adverse environments. A prominent example is the heat shock response that is triggered by exposure to elevated temperatures [1]. In this case the impaired protein folding induced by heat stress is, at least partially, counteracted by an increased expression of chaperone proteins. These assist with protein folding and, thereby, help to keep the cellular protein machinery functional in spite of adverse thermal conditions. While certain aspects of stress-elicited adaptive programs such as the heat shock response or the oxidative stress response are very well studied, the contribution of other cellular pathways including endocytosis to cellular adaptation is still elusive.

The plasma membrane can be envisioned as a central compartment in the cellular adaptation to diverse stress conditions as it shapes the interactions between cells and their environment by harboring an elaborate complement of transmembrane proteins, e.g. transporters, channels, receptors, or adhesion proteins. These cell surface proteins impinge on the vast majority of all cellular functions by mediating nutrient uptake, preserving ion homeostasis and initiating complex signaling cascades in response to extracellular cues. Consequently, the dynamic remodeling of the cell surface proteome is likely a crucial process in the cellular adaptation to many stress conditions.

A powerful pathway for rapidly altering the protein composition of the plasma membrane is Clathrin-mediated endocytosis (CME). In CME, Clathrin together with additional endocytic factors causes the invagination of a plasma membrane patch into a 100 nm-sized vesicle that delivers its cargo to the endosomal system [2]. This pathway was proposed to account for ~95% of total protein endocytic flux [3] and, thus, is the prime candidate for modulating the surface proteome upon stress. CME is especially well suited to this task by allowing the selective uptake of specific transmembrane proteins via the recognition of distinct sorting motifs by endocytic adaptor proteins. In fact, different reports have highlighted that there is close crosstalk between endocytosis, early endosomal trafficking and cellular stress.

In this review we summarize reported stress-induced adaptive changes in the surface proteome that may be brought about by alterations in CME or downstream endocytic trafficking and discuss how these might contribute to cellular survival.

## MECHANISM OF CME

CME is initiated by the recruitment of early-acting endocytic proteins, termed adaptors, such as FCHo, Eps15, Eps15R, and the Clathrin assembly protein complex 2 (AP-2) as well as curvature-inducing proteins such as Epsins and CALM to the plasma membrane [2]. These adaptors link Clathrin to the underlying membrane via their association with charged plasma membrane lipids and couple the assembly of the Clathrin coat with the selection of transmembrane cargo proteins, e.g. receptors and their ligands.

Endocytic adaptors are crucial for the endocytic process as they conduct the selection of membrane proteins destined for endocytosis (i.e. cargo) [4]. For example, specific adaptors such as ARH enable liver cells to internalize low-density lipoprotein receptors (LDLRs) to clear cholesterol from the circulation. Loss of these adaptors causes hypercholesterolemia and atherosclerosis in humans [5]. Other examples include β-arrestins that sort active G-protein coupled receptors (GPCRs), Epsins, Eps15 and Eps15R that recognize ubiquitinated receptors [6], and CALM and AP180 which mediate the endocytosis of so-called SNARE proteins that are crucial for membrane fusion [7, 8]. In addition, many internalized transmembrane proteins are recognized by the AP-2 complex, a heterotetramer of two large (α, β2) and two small subunits (μ2, σ2), that acts as a more general cargo adaptor. AP-2 harbors specific sites for its association with dileucine- (i.e. [DE]XXXXL[LI], X = any amino acid) and tyrosine-based (i.e. YxxØ, X = any amino acid, Ø = large hydrophobic residue) endocytic sorting motifs [9–11] as well as for basic C2 domains [12]. The latter are also recognized by the AP-2µ related adaptor proteins Stonin2 [13] and SGIP1 [14].

Cargo selection is closely coordinated with the assembly of the Clathrin lattice to generate productive endocytic structures. AP-2 in this mechanism acts as a co-incidence detector by initially adopting a closed conformation in which all but one membrane binding site as well as its cargo and Clathrin binding sites are occluded [15, 16]. This inactive conformation prevents the association of AP-2 with cargo proteins at sites other than the plasma membrane. The switch to the active open conformation is promoted by the coincident binding of AP-2 to the plasma membrane lipid phosphatidylinositol(4,5)bisphosphate (PIP_2_) and the AP-2 activating domain of FCHo proteins [17]. In the open conformation further PIP_2_ binding sites are exposed, and the interaction surfaces for cargo and Clathrin become accessible. Cargo, PIP_2_, and Clathrin binding stabilize the open conformation. Phosphorylation of the µ2 subunit by the adaptor associated kinase 1 (AAK1) or GAK [18, 19] promotes the open conformation, whereas NECAPs convert the phosphorylated open conformation of AP-2 into its inactive closed form [20, 21]. Hence, AP-2 is a central target for regulation in CME. One of the physiological triggers for AP-2µ phosphorylation is for example dopamine. Dopamine binding to its cognate GPCR leads via the activation of AAK1 and PKC-ζ to AP-2µ phosphorylation, thereby promoting endocytosis, e.g. of the Na^+^/K^+^-ATPase [22].

As cargo selection and concomitant Clathrin assembly progress, the assembled coat and the underlying membrane bend inward, into the direction of the cytoplasm, until eventually a vesicle-like structure is formed, the endocytic pit, which remains connected to the membrane only via a narrow stalk [2]. Active membrane remodeling during this transition from a flat Clathrin lattice to a deeply invaginated endocytic pit is facilitated by curvature-sensing and -inducing Bin-Amphiphysin-Rvs (BAR) domain-containing proteins such as FCHo, Sorting Nexin 9 (SNX9), Amphiphysin, and Endophilin. Finally, the membrane stalk is fissioned by the GTP hydrolyzing mechano-chemical enzyme Dynamin [23], and the Clathrin coat is shed with the help of the chaperone Hsc70 and auxilin or GAK [24] resulting in the release of a cargo-filled vesicle into the cytoplasm. The neuronal isoform 1 of Dynamin and FCHSD2, a protein that promotes actin polymerization at the base of Clathrin-coated pits [25], were both shown to be phosphorylated as downstream targets of oncogenic signaling cascades that stimulate endocytosis [26, 27] making it likely that they also constitute regulatory points during stress.

During the late stages of endocytosis the forming endocytic vesicle is primed for its fusion with early endosomes via the activation of the small GTPase Rab5 at the onset of vesicle uncoating [28]. Rab5-containing endosomes act as sorting platforms for cargo delivery either to a recycling pathway or for lysosomal degradation. Consistently, loss of function of Rab5 has been shown to impair endocytosis [28, 29] and to inhibit early endosome fusion [30]. The balance between the delivery of newly synthesized membrane proteins, their endocytosis, recycling and degradation will determine the pool of membrane proteins present at the plasma membrane. Therefore, alterations in endocytosis constitute a rapid means of altering the surface levels of select proteins and identify CME as an attractive mechanism for the acute cellular adaptation to stressful environmental changes.

## MECHANISMS OF REGULATION OF CME UPON STRESS CONDITIONS

**Figure 1:**
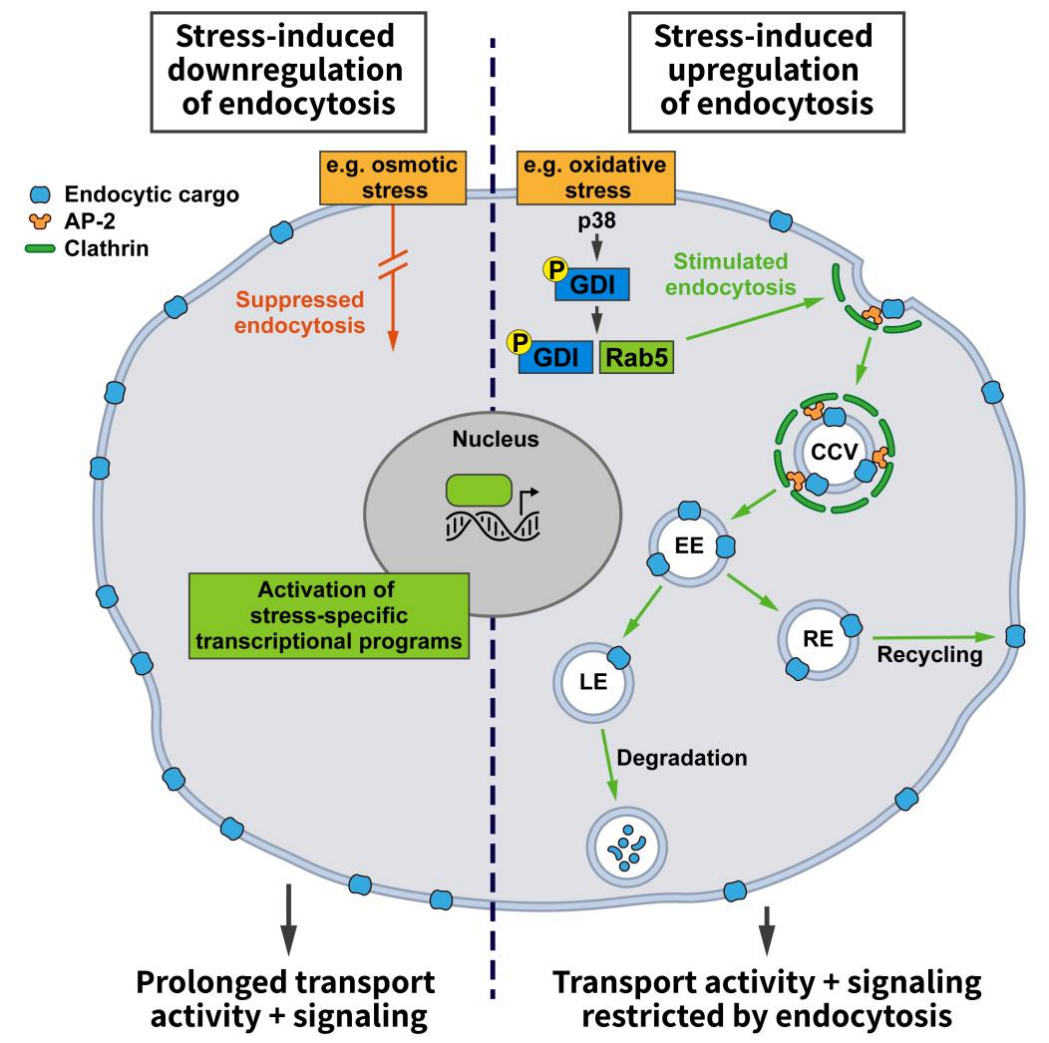
FIGURE 1: Effects of stress conditions on Clathrin-mediated endocytosis. Stress conditions can either promote (right side) or hamper (left side) endocytosis. Increased endocytosis, e.g. triggered by the stress-induced activation of the mitogen-activated protein kinase p38 that activates Rab5 by promoting the formation of GDI:Rab5 complexes, reduces the cell surface levels of transmembrane cargo proteins. This may, for example, restrict signaling from the plasma membrane or the transporter-mediated uptake of nutrients and/or enhance the endocytic uptake of specific protein-bound ligands. Conversely, downregulation of endocytosis will extend the residence time of signaling receptors and transporters at the cell surface, thereby promoting their activities (see text and other figures for details).

Adaptive programs elicited by environmental stresses such as starvation, hypoxia, or alterations in ion strength or composition, among many other challenges, could either regulate bulk endocytic flux or alter the uptake of select proteins. Altering bulk endocytic flux would result in a non-selective increase or decrease in the endocytic uptake of various cargos. This might have general benefits, for example, energy preservation when overall endocytosis is downregulated or the restoration of membrane tension when endocytosis is upregulated. An increase in endocytosis during stress might also be beneficial to elevate nutrient intake and thereby strengthen cellular resilience (**Figure 1**). For example, iron, which is taken up via CME of transferrin, is an important cofactor for a range of cellular enzymes including those required for mitochondrial oxidative phosphorylation [31]. To induce an overall change in endocytosis, key players in CME such as Clathrin, AP-2, or Dynamin may be altered in their activities or levels.

Stress-induced changes in the endocytosis of select membrane proteins on the other hand could be used to switch on or to amplify specific cellular pathways counteracting deleterious stress effects. To achieve a selective change in the endocytosis of a specific membrane protein, the first option would be to modify the protein itself to alter its affinity for the endocytic machinery. These modifications are normally posttranslational. For example, proteins can be marked for endocytosis via phosphorylation. This is the case for GPCRs that following phosphorylation by G-protein coupled receptor kinases (GRKs) bind to their specific endocytic adaptor, β-arrestin. Alternatively, cargo proteins can become ubiquitinated like the epidermal growth factor receptor (EGFR) or vascular endothelial growth factor receptor 2 (VEGFR2) for sorting by endocytic adaptors such as Eps15, Eps15R and Epsins that carry ubiquitin interaction motifs. Alternatively, modifications of specific adaptor proteins might be employed to trigger changes in the endocytosis of select cargos. For example, ARH, the endocytic adaptor that facilitates the internalization of ligand-bound LDLRs, needs to be S-nitrosylated to interact with AP-2 [32]. This type of modification could conceivably be modulated by cellular redox state and, thus, be responsive to certain types of stress. Since a number of kinases such as protein kinase N (PKN) and the mitogen-activated protein kinase (MAPK) p38 are known to be sensitive to cellular stress conditions, such as changes in osmolarity, it is also conceivable how stress-induced signal cascades could lead to alterations in the uptake of specific proteins by introducing the phosphorylation of cargo or adaptor proteins.

In fact, the p38 pathway is one of the few stress-responsive signaling cascades that have been linked to alterations in endocytosis. Among other targets, the activation of this pathway was shown to alter the activity state of Rab5 [33], which is not only crucial for early endosomal membrane traffic, but is also known to affect endocytosis [29]. Rab5 in its cytosolic GDP-bound state is complexed to guanyl-nucleotide dissociation inhibitor (GDI), which promotes its extraction from membranes. GDI activity is stimulated by active p38, and the increase in Rab5:GDI complexes appears to promote endocytosis in line with earlier reports [28]. Even though this study only analyzed the uptake of HRP and dextrans [33], i.e. signature cargos of fluid phase endocytosis, it is likely that the findings can be extended to CME since Rab5:GDI has been proposed to act at Clathrin-coated pits [28].

Given the multitude of different environmental stresses and their potential impact on distinct cell and organ systems, it is not surprising that no systematic studies on the regulation of CME by various stress conditions have been performed and no consensus picture or mechanism has emerged yet. Moreover, in many studies, specific effects of environmental stresses on the endocytosis or plasma membrane level of a defined membrane protein have not always been discerned from effects on CME in general. Hence, it often remains unclear whether a given stress elicits a cargo-selective endocytic change or a more global effect on CME and/or associated pathways, e.g. endosomal sorting downstream of CME. We have therefore refrained from a clear distinction between these mechanisms. Instead, we provide an overview of the various reports describing a regulatory role of different stress conditions on the endocytosis of distinct membrane proteins (**Table 1**).

**TABLE 1.** Effects of different stress conditions on the endocytosis of distinct membrane proteins.

**STRESS**	**EFFECT ON ENDOCYTOSIS**	**CARGO**	**REFERENCE**
NUTRIENT DEPRIVATION	REDUCED	Dextran, arginine transporter	[35], [120]
	ENHANCED	α5β1 integrins, β4 integrins, SNAT2, SNAT3, Serine Incorporator 1, Death receptors 4, Death receptors 5	[38], [121], [122]
GLUCOSE DEPRIVATION	REDUCED	GLUT1	[39]
	ENHANCED	Uracil permease, amino acid permease, GluA2, GluA3, GABA_A_R	[40], [41], [42]
HYPERTONICITY	REDUCED	[3H]f-NleLeuPhe, [4C]sucrose, hemocyanin, dextran, transferrin, FM dye, aquaporin2, GLUT4, NHE7	[48], [50], [51], [53–55], [123]
	ENHANCED	EGFR	[104], [115]
HYPOTONICITY	REDUCED	Dextran, transferrin, β-VLDL	[44–46]
	ENHANCED	Dextran, IgG-latex beads, Claudin-1, Claudin-2	[63], [64]
SHEAR	ENHANCED	LDL, FVIII-VWF, albumin, dextran	[65–69], [71], [72]
HYPOXIA	REDUCED	Na^+^/K^+^-ATPase	[79]
	ENHANCED	Na^+^/K^+^-ATPase, ENaC, GLR-1, EGFR	[77], [80], [81], [124]
HYPERCAPNIA	ENHANCED	Na^+^/K^+^ATPase, ENaC	[82], [125]
HYPOCAPNIA	ENHANCED	Na^+^/K^+^ATPase	[84]
ROS	REDUCED	LDL, transferrin, EGFR, lectin receptor, GLUT4, aquaporin 4	[85–93]
	ENHANCED	Multidrug resistance-associated protein 2, bile salt export pump	[95–98]
IRON DEFICIENCY	REDUCED	DMT1, TfR	[100], [102]
IRON OVERLOAD	ENHANCED	DMT1	[101]
CISPLATIN	ENHANCED	EGFR	[104]
HEAVY METALS	REDUCED	Dextran, albumin	[103], [104], [106], [107],
IRRADIATION	REDUCED	Dextran, lucifer yellow	[111], [113]
	ENHANCED	EGFR	[114], [115]

## STRESS CONDITIONS THAT AFFECT ENDOCYTOSIS

In contrast to the molecular mechanism of CME that has been dissected in detail during the past decades, the regulation of CME under different physiological conditions remains poorly understood. It is generally assumed that endocytosis is a constitutive process. However, different cellular and environmental factors can influence the efficiency of endocytosis. Alternatively, as alluded to above, environmental stresses may impact on the endocytosis or plasma membrane levels of specific membrane proteins. In this chapter we will summarize what is known with respect to various types of stress conditions and their impact on endocytosis.

### Oncogenic signaling

The most systematically addressed regulatory influence on CME so far is oncogenic signaling by which tumor cells “adapt” CME for their purposes [27]. By screening a panel of oncogenic signaling kinase inhibitors, the Schmid lab uncovered that ten of the 21 studied pathways affected CME [27], even though for most of them it is still unclear how they influence CME mechanistically. Concentrating on ERK1/2 signaling, Xiao *et al.* showed that ERK-dependent phosphorylation of FCHSD2, which acts in the maturation of Clathrin-coated pits [25], promotes FCHSD2 recruitment to the plasma membrane and, thereby, CME [27]. Thus, aberrant ERK signaling appears to be a crucial factor for the rapid CME observed in cancer cells. While the initial screening was based on transferrin uptake, subsequent experiments demonstrated that increased CME also leads to elevated EGFR uptake. This appears at first glance counterintuitive, given that increased receptor tyrosine kinase signaling is generally considered beneficial for oncogenesis. However, as EGFR signaling is complex and takes place at both, the plasma membrane and early endosomes, the net signaling outcome in a given type of cancer cell is hard to predict. In fact, while a number of cancer cells show ERK- and FCHSD2-dependent upregulation of endocytosis, in non-small-cell lung cancer (NSCLC) loss of function of FCHSD2 has been suggested to promote cell proliferation and migration [27]. The complex effects of altered CME capacity on oncogenesis may depend on the tumor cell type as reflected by the fact that high FCHSD2 levels are correlated with increased survival in NSCLC [27], whereas elevated FCHSD2 may hamper survival in acute myeloid leukemia [34]. Thus, depending on concomitant signaling changes in cancer cells, further oncogenesis may be driven either by a down- or upregulation of endocytosis. The differential regulation of CME in cancer cells might also reflect the fact that pro- and anti-oncogenic signaling pathways converge onto CME factors and may differentially adapt CME to cellular needs. Analogous pathways likely exist for the adaptation of CME to other types of stress conditions.

### Nutrient and energy deprivation

#### Endocytosis and nutrient signaling

**Figure 2:**
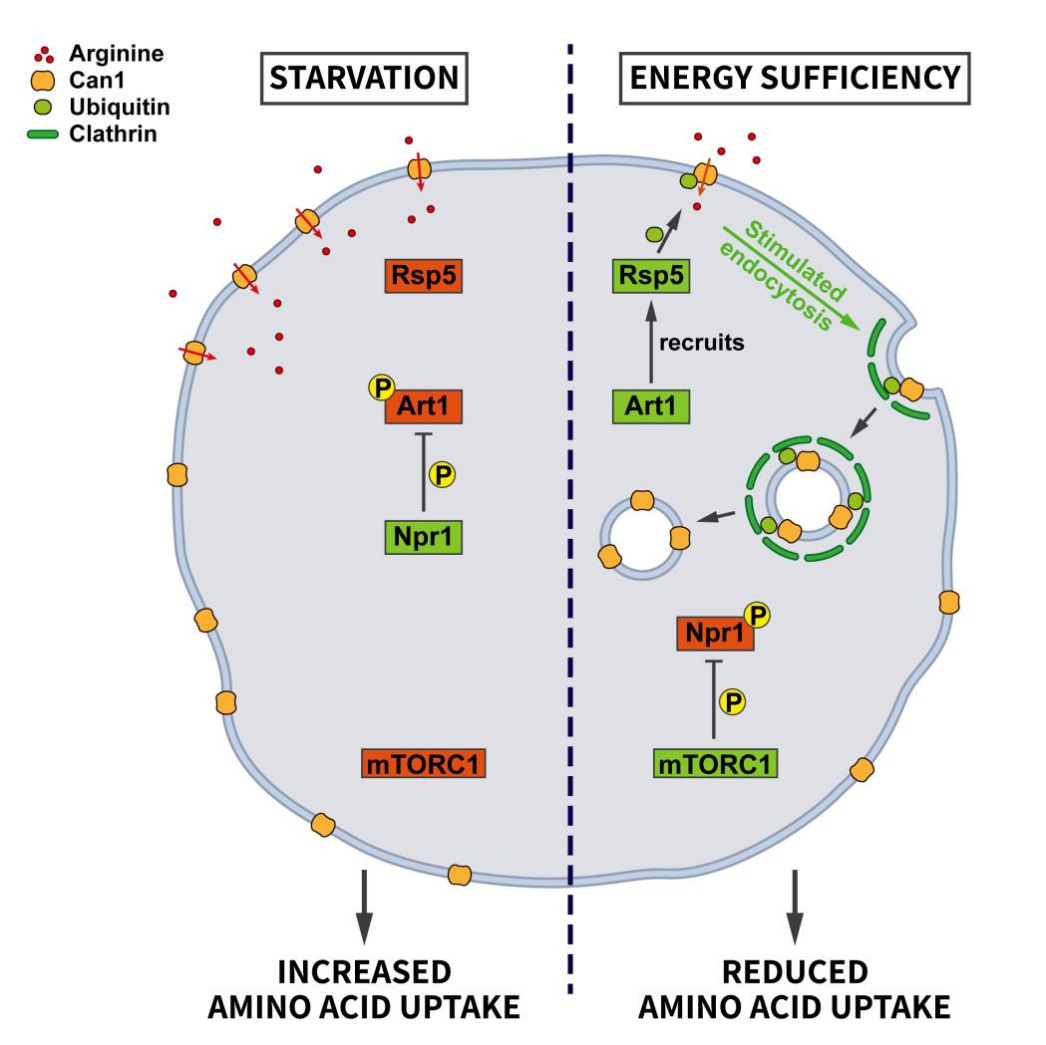
FIGURE 2: Regulation of amino acid transporter endocytosis by nutrient stress. During energy sufficiency the arrestin-like adaptor Art1 recruits the ubiquitin ligase Rsp5 to the plasma membrane to induce the ubiquitination of the arginine transporter Can1. Subsequent endocytosis of Can1 limits amino acid uptake. This pathway is regulated by the central nutrient sensor mTORC1 which in its active state keeps the negative regulator of Art1, the kinase Npr1, inactive. During starvation mTORC1 is rendered inactive so that Npr1 can inhibit Art1 via phosphorylation. Consequently, Can1 is not ubiquitinated by Rsp5 and remains at the plasma membrane to increase arginine uptake (see section ”Endocytosis and nutrient signaling” for further details). CCV, clathrin-coated vesicle; EE, early endosome; RE, recycling endosome; LE, late endosome.

Endocytosis provides cells with nutrients. Hence, it is plausible that control of the endocytic pathway should be sensitive to nutrient availability. However, the role of endocytosis in nutrient supply and, conversely, the regulation of endocytosis by nutrients appears to be complex. Work in yeast has revealed an elegant adaptive response by which nutrient deprivation not only activates autophagy for amino acid recycling, but also inhibits the endocytosis of specific amino acid transporters, e.g. the arginine transporter Can1, to promote amino acid uptake [35]. This mechanism is regulated by nutrient signaling via mTORC1, the arrestin-like adaptor Art1, the ubiquitin ligase Rsp5, and the kinase Nrp1 (**Figure 2**). At steady state, Can1 is ubiquitinated by the ubiquitin ligase Rsp5, which promotes Can1 endocytosis. The ubiquitin ligase adaptor Art1 is necessary to target Rsp5 to Can1. Art1 is a substrate for the kinase Npr1, which phosphorylates Art1 and, thereby, causes its inactivation by limiting its plasma membrane association. Under conditions of energy sufficiency Npr1 is repressed by active mTORC1, thereby enabling Art1 to trigger the ubiquitina-tion-dependent endocytosis of Can1. During starvation when mTORC1 activity is repressed, Npr1 becomes active, resulting in Art1 inhibition and a blockade of Can1 ubiquitination and endocytosis to promote the uptake of amino acids. This work thus suggests a mechanism whereby mTORC1 promotes the internalization of amino acid transporters upon high nutrient availability, whereas under starvation decreased mTORC1 activity shuts down their endocytosis [35].

Art1 belongs to a family of arrestin-related proteins [36]. Therefore, it is conceivable that the outlined mechanism might encompass even more cargo proteins via the stress-dependent regulation of additional Art proteins. It is not clear yet whether this pathway also operates in mammalian cells. However, mammalian cells express a family of arrestin-domain-containing proteins that are known to link cargo proteins to ubiquitination. Although there is no obvious homolog of Npr1 in mammalian cells, it is interesting to note that it belongs to the same kinase family as the mammalian AMP-activated kinase (AMPK). AMPK has recently been shown to be inhibited by mTORC1 [37] and might therefore be a possible candidate for the reported mTORC1-dependent downregulation of endocytosis [35].

It is likely that the repression of Can1 internalization in nitrogen-starved yeast cells is a cargo-specific response rather than reflecting a general repression of endocytosis upon nutrient deprivation, since other studies propose that starvation rather favors endocytosis, conceivably to enhance protein degradation and, thereby, increase nutrient availability to maintain cell function. A recent study in mammalian cells combining SILAC with mass spectrometry reported that around 3% of the proteome is degraded following starvation [38]. Many of the substrates found were plasma membrane receptors that are degraded via endocytosis and subsequent lysosomal proteolysis to protect from starvation-induced cell death.

#### Glucose and energy deprivation

**Figure 3:**
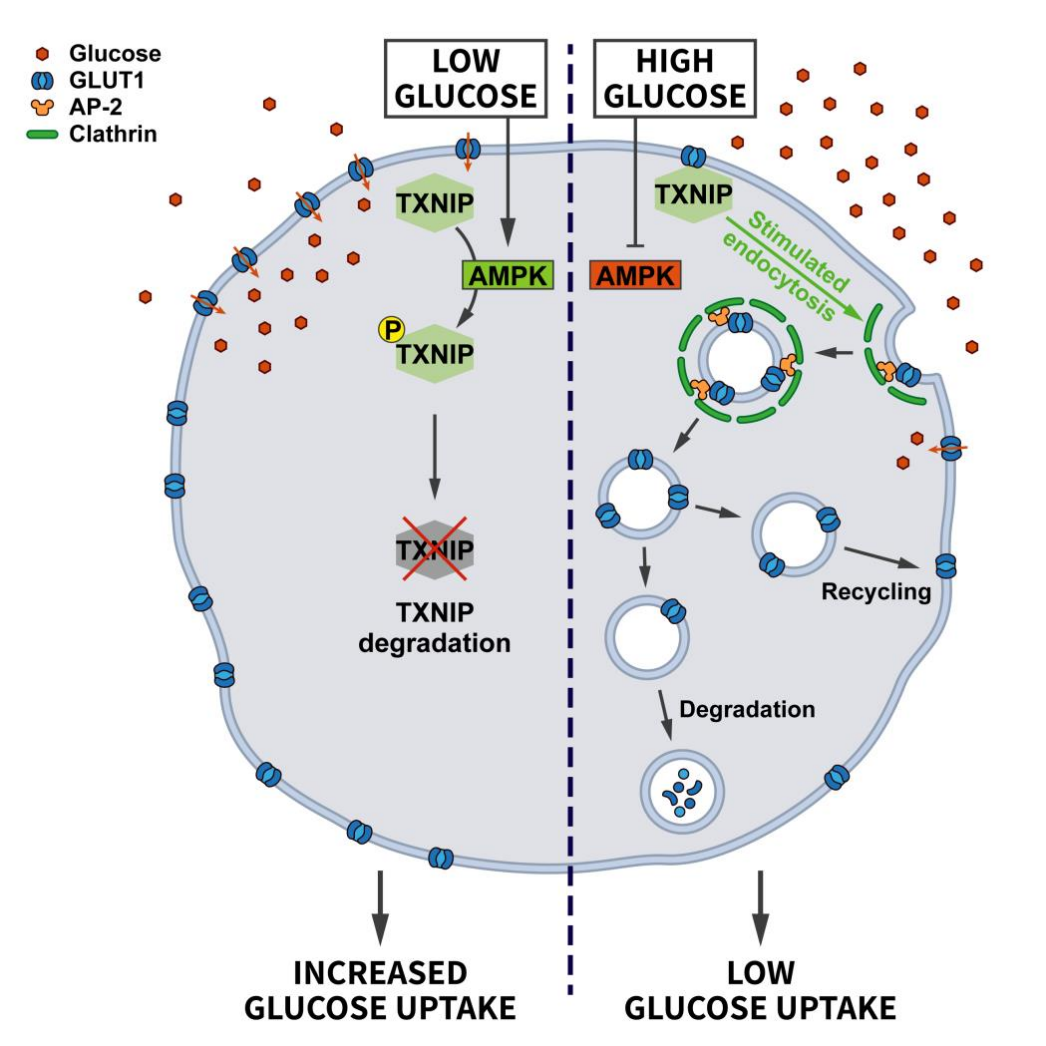
FIGURE 3: Regulation of glucose transporter endocytosis by nutrient stress. Under conditions of abundant glucose the adaptor protein TXNIP promotes the endocytosis of GLUT1 to restrict glucose uptake. When glucose levels decline, AMPK activation triggers the degradation of TXNIP, thereby downregulating GLUT1 endocytosis to promote glucose uptake (see section ”Glucose and energy deprivation” for further details).

Acute cellular energy deprivation requires adaptive changes such as increased uptake of glucose. Most cells take up glucose via the glucose transporter GLUT1, a membrane protein regulated by CME. At steady state the surface levels of GLUT1 are kept in check by the arrestin-domain containing protein TXNIP, which promotes the internalization of GLUT1. When ATP is running low and the AMP/ATP ratio increases, AMPK gets activated and phosphorylates TXNIP, thereby inducing its degradation. As a consequence, the endocytosis of GLUT1 is repressed and glucose uptake is promoted to relieve energy stress [39] (**Figure 3**).

Endocytosis of non-essential proteins indeed may assist survival under conditions of limited energy supply. In yeast, low glucose levels promote the endocytosis of plasma membrane proteins, such as the uracil permease Fur4 and the hydrophobic amino acid permease Tat2, to the vacuole/lysosome, while recycling to the surface is inhibited, thereby promoting endocytic flux to the vacuole [40]. The mechanism that renders the recycling pathway sensitive to glucose starvation remains incompletely understood.

In the brain, impaired energy metabolism is linked to ischemia and to neurodegenerative diseases. Oxygen and glucose deprivation (OGD) invoke pathogenic processes such as increased glutamate excitotoxicity, Ca^2+^ overload, mitochondrial impairment, oxidative and endoplasmic reticulum stress, inflammation, and apoptosis explaining the ischemia-induced cerebral injury. Hypoxia inducible factors (HIFs) and the AMPK signaling pathway are known to be involved in the response to OGD. Moreover, endocytosis of the ionotropic glutamate receptors GluA2 and GluA3 in rat hippocampal neurons has been proposed to contribute to excitotoxic neuronal cell death following OGD, as more Ca^2+^-permeable glutamate receptors such as GluA1 homomers remain on the neuronal surface under these conditions [41]. How GluA1 endocytosis upon OGD is prevented and why endocytosis of GluA2/3 happens specifically in hippocampal, but not in cortical neurons is unclear. Likewise, AP-2 mediated-internalization of GABA_A_ receptors from the surface of dendrites, via recognition of a specific AP-2 binding motif in the GABA_A_ receptor β3 subunit, is promoted during OGD. Blocking OGD-associated GABA_A_ receptor endocytosis therapeutically to safeguard GABA_A_ receptor surface levels and, thereby, inhibitory neurotransmission would likely be beneficial for limiting OGD-induced cell death [42].

### Osmotic stress

The control of osmotic homeostasis is crucial for a multitude of cellular functions. Consequently, cells have established various mechanisms for counteracting disturbances in cell volume. On the one hand, adaptive responses following osmotic swelling or shrinkage activate signaling pathways that initiate regulatory volume programs, which allow cells to recover their initial volume. On the other hand, mechanisms are triggered that help cells to cope with the impairments associated with changes in ionic strength such as protein aggregation. If swelling or shrinkage persist without adaptation, cell death is caused. Impaired volume regulation is associated with several pathologies such as ischemia/reperfusion, hypovolemia, hypernatremia and diabetic shock [43].

Hypotonic extracellular medium will cause cell swelling and an increase in membrane tension, while hypertonic conditions induce cell shrinkage and a decrease in membrane tension. Given that endocytosis in essence is a plasma membrane remodeling mechanism, conditions that alter plasma membrane tension are expected to affect the efficiency of internalization. Elevated membrane tension makes it harder to deform the membrane and, hence, hypotonic conditions oppose endocytosis. Consistently, impaired endocytosis of transferrin, β-VLDL, or dextrans under hypotonic conditions has been reported in various mammalian cell types [44–46]. Hypotonic stress can be counteracted to some degree by the actin cytoskeleton such that endocytosis persists even under conditions of increased membrane tension [47]. Based on these considerations, hypertonic conditions would be expected to facilitate endocytosis. However, that does not seem to be the case as demonstrated more than 40 years ago [48]. Elegant electron microscopic studies revealed that hypertonicity leads to the formation of abnormal membrane-free Clathrin microcages, thereby preventing the assembly of normal Clathrin-coated vesicles. This might be due to the fact that cell shrinkage is accompanied by cytosolic acidification, a condition known to trigger Clathrin assembly [49]. Additional factors, such as the increased concentration of proteins and divalent cations in the shrunken cells, might also contribute. Decreased receptor endocytosis upon hypertonicity has been confirmed in a variety of cell types such as polymorphonuclear leukocytes [48], *Dictyostelium* [50] and type I alveolar epithelial cells [51]. In addition, some studies also found fluid-phase endocytosis to be affected under osmotic shock [50, 51]. Hence, hypo- and hypertonic stress both impair CME.

An important trigger for plasma hyperosmolarity is elevated blood glucose (hyperglycemia). Like insulin, hyperosmolarity causes a reduction in the AP-2-dependent [52] endocytosis of GLUT4, thereby elevating its surface pool. This osmoadaptive mechanism contributes to the clearance of glucose from the blood [53]. Similarly, in epithelial renal cells aquoporin2 (AQP2) displays elevated cell surface levels upon hyperosmotic shock [54], mainly due to lower rates of AQP2 endocytosis. This adaptation will promote transcellular water flux that might be beneficial for surviving under severe hypertonic stress.

**Figure 4:**
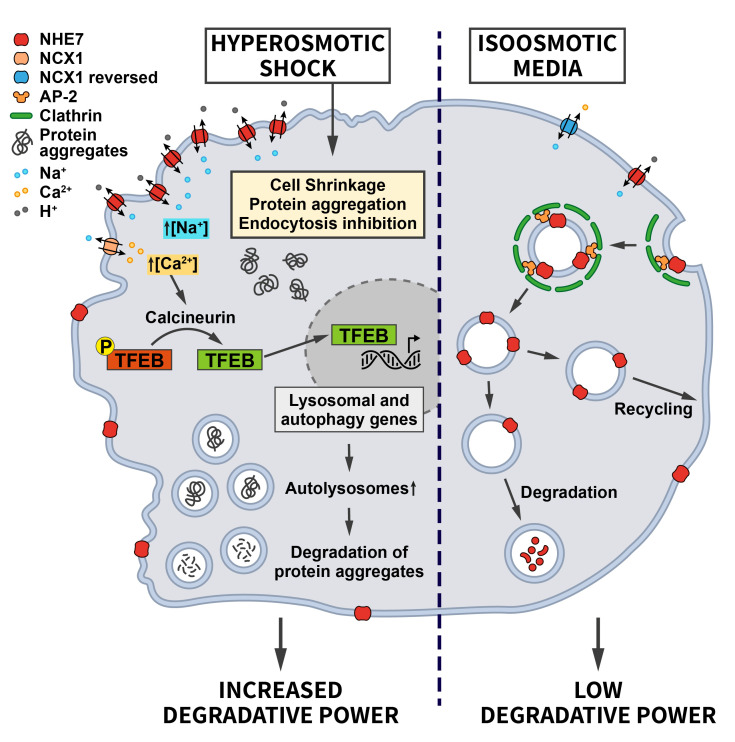
FIGURE 4: Regulation of ion transporter endocytosis by osmotic stress. Under iso-osmotic conditions NHE7 is continuously endocytosed via CME, thereby limiting its transport activity at the cell surface. Upon hyperosmotic conditions, endocytosis of NHE7 is downregulated resulting in elevated NHE7 surface levels. Increased NHE7 activity at the plasma membrane elevates Na^+^ influx, which via the Na^+^/Ca^2+^ exchanger NCX1 leads to increased intracellular Ca^2+^ levels and the Ca^2+^/Calcineurin-mediated dephosphorylation of the transcription factor TFEB to induce lysosomal and autophagy gene expression. The ensuing increased cellular degradative capacity is beneficial for counteracting protein aggregation caused by hyperosmotic conditions (see section ”Osmotic stress” for further details).

Most recently, our laboratory discovered that the differential endocytic sorting of the intracellular Na^+^/H^+^ exchanger NHE7 is important for coping with hypertonic stress [55]. Whereas NHE7 under isotonic steady-state conditions primarily resides on endosomes and the trans-Golgi-network, it is redistributed to the cell surface during the early events associated with hyperosmotic shock. A similar redistribution of NHE7 is observed when CME is impaired, suggesting that NHE7 is constitutively endocytosed. The surface enrichment of NHE7 upon hyperosmotic stress is beneficial for cell survival: Plasma membrane NHE7 via downstream changes in cellular ion homeostasis and the resulting activation of the transcription factors TFEB/TFE3 promotes autophagosome and lysosome biogenesis to potentiate the degradative capacity of osmotically stressed cells. The osmotic stress-induced activation of the NHE7-TFEB pathway enables cells to cope with the increased load of aggregated proteins that accumulate during the osmotic insult and, thereby, promotes cellular survival (**Figure 4**). How CME of NHE7 is repressed upon hypertonic stress at the mechanistic level requires further investigations.

The NHE7-TFEB/TFE3 mechanism likely contributes to the observed induction of autophagy triggered by hypertonic stress [56, 57] and may synergize with the increased synthesis of phosphatidylinositol-3,5-bisphosphate, a lipid crucially required for lysosomal proteolysis, in yeast [58, 59] and mammalian cells challenged with hyperosmotic conditions [60]. These examples illustrate how alterations in endocytosis and adaptations within the autophagy/endolysosomal system downstream of endocytosis may play a key role in the intracellular anti-stress response.

Studies in yeast have added some insight into the possible mechanism underlying osmotically induced changes in endocytosis. Hypertonicity was reported to trigger alterations in intracellular Ca^2+^, thereby activating the Ca^2+^/Calmodulin–dependent phosphatase Calcineurin. Calcineurin dephosphorylates and activates the endocytic lipid phosphatase Synaptojanin/Inp53, thereby promoting endocytosis to compensate the excess of membrane caused by hypertonicity [61]. A similar Calcineurin-dependent bulk endocytic response can be triggered at synapses of mammalian neurons by sustained high-frequency firing activity [62].

Interestingly, changes in tonicity have been suggested to induce endocytosis in cells of the immune system to regulate inflammatory responses. For example, hypotonic stress was reported to promote the immune function of macrophages by boosting their endocytic ability [63]. The chloride channel ClC-3 was implicated in this pathway, however, the underlying mechanism is presently unclear. In contrast, the endocytosis of claudin-1 and -2 in epithelial tubular cells in the kidney upon osmotic stress caused by alterations in the osmolarity of the tubule fluid [64] rather represents a detrimental response, since breakdown of tight junctions and the ensuing paracellular transport accelerate renal cell injury.

### Mechanical Stress

Other types of mechanical stress aside from changes in membrane tension induced by altered osmolarity have also been shown to affect endocytosis. The best example is shear stress that is experienced by cells lining vessel walls such as endothelial cells. The uptake of LDL into endothelial cells is increased under conditions of shear stress [65–68]. Interestingly, LDLR family members such as LRP1 also contribute to the shear stress-induced uptake of a complex between the coagulation factor VIII (FVIII) and its carrier protein von Willebrand factor (VWF) into macrophages [69].

**Figure 5:**
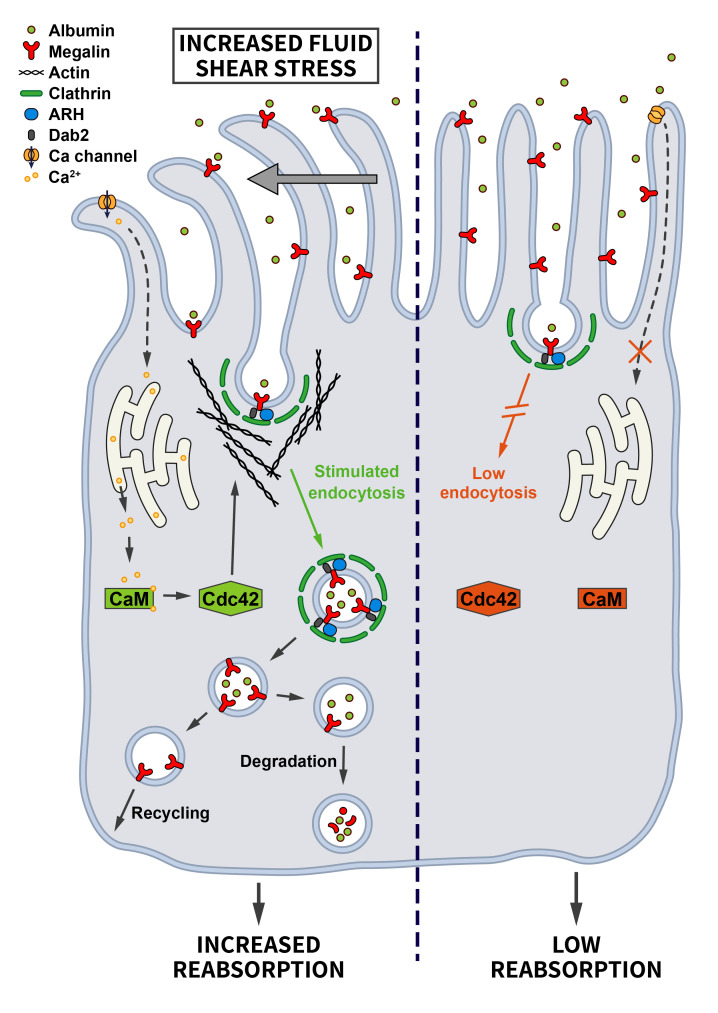
FIGURE 5: Regulation of endocytosis by mechanical stress. Proximal tubule cells are tasked with the uptake of proteins and other molecules from the glomerular filtrate. Increased fluid shear stress bends primary cilia, thereby triggering Ca^2+^ influx that is further amplified intracellularly. Via activation of Calmodulin this change induces Cdc42-mediated actin polymerization and enhanced endocytosis to prevent proteinuria (see section ”Mechanical stress” for further details).

In the kidney, cells sheathing the proximal tubule are highly specialized in retrieving proteins and other small molecules from the glomerular filtrate, and impaired endocytosis is known to contribute to the pathogenesis of several renal diseases. To adapt their uptake capacity to changes in glomerular filtration rate, proximal tubule cells are responsive to shear stress. In fact, the shear stress induced by the flow of the glomerular filtrate determines the extent of endocytosis on the lumenal side of proximal tubule cells [70, 71]. The important role of CME in proximal tubule-derived epithelial cells exposed to shear stress was confirmed by demonstrating that increased shear stress leads to a ~2-fold increase in the Clathrin-dependent uptake of albumin and dextran via the multiligand receptors Megalin and Cubilin. This pathway involves the shear-stress induced bending of cilia that may trigger Ca^2+^ influx via mechanosensitive channels and Ca^2+^ release from the endoplasmatic reticulum downstream of ATP-activated purinergic P2YR receptors. Elevated cytosolic Ca^2+^ via Calmodulin induces the activation of Cdc42 [72], which by promoting actin polymerization may facilitate endocytosis (**Figure 5**). In fact, actin is known to be important for endocytosis at the apical side of polarized cells to overcome membrane tension [47]. This appears to be a very cell-type specific pathway that only operates in cells of proximal tubule origin [71].

### Stress due to imbalances in O_2_/CO_2_ supply and oxidative species

#### Hypoxia – O_2_ stress

**Figure 6:**
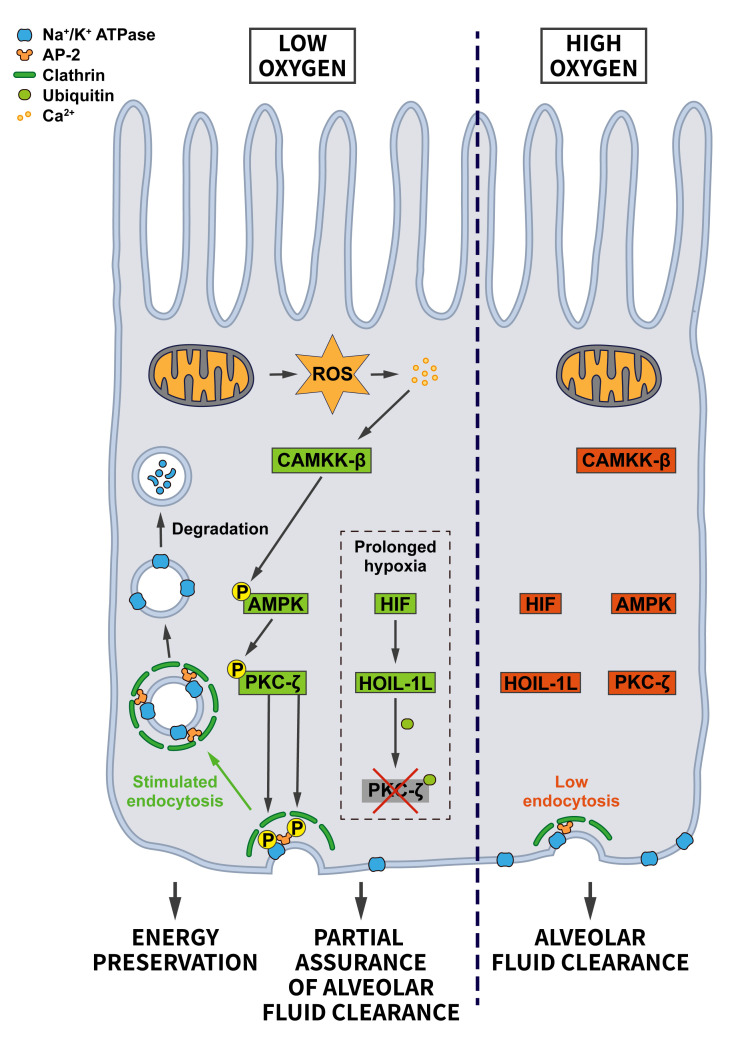
FIGURE 6: Regulation of endocytosis by oxygen stress. Under adequate oxygen supply there is limited endocytosis of the Na^+^/K^+^-ATPase, which is needed at the plasma membrane to build up the ion gradient that supports alveolar fluid clearance. When oxygen levels drop, the endocytosis of Na^+^/K^+^-ATPase is promoted to preserve energy. Hypoxia-induced ROS via an increase in Ca^2+^ lead to the activation of CAMKK-β which activates AMPK and PKC-ζ. PKC-γ phosphorylates sites on AP-2 and the Na^+^/K^+^-ATPase to boost Na^+^/K^+^-ATPase endocytosis. During prolonged hypoxia, the HIF pathway triggers the ubiquitination and degradation of PKC-ζ by elevating the expression of the E3 ubiquitin ligase HOIL-1L. In this way cells balance energy preservation and alveolar fluid clearance for cellular and organismal survival (see section ”Hypoxia – O_2_ stress” for further details).

Hypoxia, inadequate oxygen supply, is a common stressor that cells face during fetal development, upon environmental variations such as high altitudes, and during pathological conditions like myocardial or cerebral ischemia, and in cancer (see ”Oncogenic signaling”) where hypoxia is very common in solid tumors [73]. Hypoxic conditions lead to the stabilization of the transcription factor HIF, which drives a transcriptional stress response for the adaptation to oxygen deprivation. Interestingly, the HIF system is also activated by oncogenes and tumor suppressor mutations [74]. In addition to triggering the HIF-mediated signaling pathway, it has been suggested that hypoxia facilitates endocytosis, thereby altering the composition of the surface proteome. For example*,* hypoxia promotes the internalization of the Na^+^/K^+^-ATPase in lung alveolar epithelium, possibly in an attempt to reduce ATP consumption [75]. CME of the Na^+^/K^+^-ATPase was shown to require recognition of a YxxØ motif in its α subunit by AP-2µ [76] and the reactive oxygen species (ROS)-induced activation of PKC-ζ, which phosphorylates both, the Na^+^/K^+^-ATPase and AP-2µ to induce Na^+^/K^+^-ATPase internalization [75] (**Figure 6**). Interestingly, hypoxia-induced Na^+^/K^+^-ATPase internalization was shown to be HIF-independent. Hypoxia also appears to facilitate the endocytosis and degradation of the alveolar epithelial sodium channel (ENaC) [77], although the underlying mechanism is less clear in this case.

The hypoxia-induced endocytosis of the Na^+^/K^+^-ATPase and ENaC are likely a cellular adaptation for preserving energy. However, a harmful consequence is the concomitant impairment of alveolar fluid clearance, which requires Na^+^ absorption via ENaC at the apical side and Na^+^ extrusion by the Na^+^/K^+^ ATPase at the basolateral membrane [78]. To limit the extent of the resulting alveolar edema, cells activate a pro-survival response that prevents excessive Na^+^/K^+^-ATPase endocytosis under severe hypoxia. Hypoxia-induced HIF signaling increases the levels of the E3 ubiquitin ligase HOIL-1L, which promotes the ubiquitination of PKC-ζ leading to its degradation. Consequently, PKC-ζ is prevented from further stimulating Na^+^/K^+^-ATPase internalization [79]. This mechanism allows cells to achieve a balance between preserving ATP and safeguarding a certain amount of alveolar fluid clearance to support survival of individual cells and the organism.

Studies in *Caenorhabditis elegans* suggest that neurons may modulate endocytosis in response to hypoxia. Hypoxic conditions or loss of activity of the cellular oxygen sensor EGL-9 facilitate endocytosis but impair the recycling of the glutamate receptor GLR-1 [80], leading to reduced GLR-1-associated currents and behavior changes. The downregulation of surface glutamate receptors triggered by hypoxia may be considered as a neuroprotective response in order to preserve neurons from glutamate receptor-mediated excitotoxicity. Whether similar pathways operate in mammalian neurons remains to be studied. These results are consistent with the downregulation of the ionotropic glutamate receptors GluA2 and GluA3 in mammalian neurons under OGD (see ”Glucose and energy deprivation”).

EGFR signaling is also over-activated under hypoxia. Low oxygen conditions prolong the EGFR half-life in a HIF-dependent manner by diminishing the rate of endosome fusion caused by reduced expression of Rab5 and its effector Rabaptin5. Rabaptin5 is normally recruited by Rab5 and stimulates the Rab5 guanine nucleotide exchange factor (GEF) Rabex5, thereby increasing Rab5 activity and early endosome fusion. Downregulation of Rabaptin5, hence, leads to a decrease of early endosome fusion and to a delay in the downstream trafficking to lysosomes and in cargo degradation [81]. Consequently, the affected receptor tyrosine kinases, which are able to signal from endosomes, display prolonged signaling responses. Prolonged receptor tyrosine kinase signaling represents an important adaptive response of the cancer cells for further oncogenesis in the hypoxic tumor environment.

#### Hypercapnia - CO_2_ stress

Hypercapnia is a condition in which CO_2_ levels in the blood are elevated, for example, due to insufficient ventilation of the alveolar epithelium, the site of gas exchange. This can occur, for example, during lung diseases such as COVID-19. Moreover, acute hypercapnic respiratory failure (AHRF) can be triggered by chronic obstructive pulmonary disease (COPD) and some forms of neuromuscular disease (e.g. myasthenia gravis and obesity hypoventilation syndrome) or any type of respiratory failure where the muscles that are needed for breathing are exhausted (e.g. severe pneumonia, acute severe asthma). To keep gas exchange optimal, alveolar epithelial cells with the help of the Na^+^/K^+^-ATPase and the Na^+^ channel ENaC normally reabsorb fluid that leaks into the airspaces [78]. However, elevated CO_2_ levels cause a Ca^2+^ influx triggering a kinase cascade involving the successive activation of CaMKK-β (Ca^2+^/Calmodulin-dependent kinase kinase-β), AMPK and PKC-ζ [78]. Thus, the signaling cascade converges here on the same kinase that is also induced in hypoxic alveolar epithelium and leads, among other changes, via AP-2 phosphorylation to increased uptake of Na^+^/K^+^-ATPase as described in section ”Hypoxia – O_2_ stress”. In addition, AMPK also triggers activation of JNK, which in turn phosphorylates the scaffolding protein Lim domain-only 7b (LMO7b). Phosphorylated LMO7b on the one hand shows enhanced colocalization with the Na^+^/K^+^-ATPase, and on the other hand may interact with AP-2µ, thereby possibly promoting the endocytosis of the Na^+^/K^+^-ATPase [82].

Another critical alveolar protein whose endocytosis is upregulated upon hypercapnia is ENaC. CO_2_ was shown to induce the ERK1/2-mediated phosphorylation of the β-subunit of ENaC. In addition, ERK1/2 causes the activation of AMPK and JNK1/2, which, in turn, phosphorylate and activate Nedd4-2. These phosphorylations lead to the Nedd4-2-dependent polyubiquitination of the β-subunit of ENaC, thereby triggering CME of the ENaC complex [83].

Akin to hypoxia, the drawback of the lack of Na^+^/K^+^-ATPase and ENaC activities under hypercapnia is the impaired alveolar fluid reabsorption that further aggravates lung injury. Therefore, preventing the CO_2_-induced downregulation of Na^+^/K^+^-ATPase and ENaC might be beneficial in conditions of lung edema. Strikingly, hypocapnia, i.e. reduced blood CO_2_ levels, was found to worsen alveolar fluid reabsorption similar to hypercapnia during lung injury by promoting endocytosis of Na^+^/K^+^ATPase [84]. Thus, opposite levels of the same stress stimulus converge on similar effects.

#### Oxidative stress

Cells experience oxidative stress when the generation of ROS such as peroxides, superoxides and free radicals is higher than their elimination. Oxidative imbalance arises either by an excess of ROS accumulation or a reduction in the amount of antioxidative enzymes. If cells encounter an excess of ROS, they need to activate programs to cope with the ROS-induced harmful changes. The stress response is turned on mainly via transcriptional activation of target genes that will either enhance survival or promote cell death.

Several studies suggest that increased oxidative stress directly or indirectly causes defects in the CME of LDL [85–89]. Altered endocytosis of other cargoes was also reported. For example, elevated ROS were shown to inhibit EGFR and lectin receptor endocytosis in fibroblasts, likely by preventing their ubiquitination [90]. Similarly, ROS were observed to impair the internalization of GLUT4 in cardiomyocytes [91], aquaporin 4 (AQP4), transferrin and LDL in astrocytes [92, 93], and of synaptic vesicle membranes in neurons [94].

Regarding the potential underlying mechanism, oxidative conditions in astrocytes were reported to lead to the downregulation of the transcription factor Sp1, which promotes the transcription of Hsc70 [93], a constitutively expressed heat shock protein and an essential cofactor for the uncoating of Clathrin-coated vesicles in CME [24]. Decreased levels of Hsc70 are expected to reduce the availability of free Clathrin for new rounds of endocytosis, since it would be sequestered in uncoating-deficient Clathrin-coated vesicles or Clathrin cages [93]. However, it is unclear whether this mechanism extends to other cell types and conditions.

On the other hand, oxidative conditions have been suggested to enhance the endocytosis of apically-located transporters in liver cells. These include the multidrug resistance-associated protein 2 (Mrp2) and the bile salt export pump (Bsep) [95–97], a protein important for canalicular bile secretion. A disorganization of the actin cytoskeleton appears to be the reason for the elevated endocytosis of Mrp2 and Bsep, most likely as a consequence of ROS-induced altered Ca^2+^ homeostasis and PKC activation. Importantly, exogenous application of bilirubin, known for its antioxidant properties, rescues the ROS-induced changes in actin disruption and Mrp2 and Bsep downregulation [97], thereby counteracting the hepatocanalicular dysfunction associated with oxidative stress. A recent study also shows similar results for Rifampicin, an antibiotic commonly used to treat tuberculosis, but with detrimental side effects like hepatotoxicity. Rifampicin leads to increased Mrp2 endocytosis by causing oxidative stress and by activating PKC-ERK/JNK/p38 and PI3K signaling pathways. Interestingly, the Rifampicin-induced elevation in Mrp2 endocytosis might also be due to transcriptional/protein upregulation of Clathrin and AP-2, changes that are suppressed by antioxidants [98].

Clearly, further studies are needed to examine the effects of ROS on endocytosis and the underlying molecular mechanisms.

### Stress caused by exposure to metals: Iron, platinum, and heavy metals

#### Iron

Endocytosis is the main route for cellular iron uptake, which is a crucial cofactor of cellular enzymes including those required for mitochondrial oxidative phosphorylation [99]. Its uptake is also associated with an increased generation of ROS, explaining why iron trafficking and metabolism must be exquisitely regulated. Iron homeostasis is under control of iron regulatory proteins (IRPs) that transcriptionally regulate the expression of target genes to maintain cellular iron metabolism such as genes encoding proteins involved in iron uptake [100].

One of the causes of iron overload is an elevated intake via intestinal absorption. In enterocytes, iron is mainly taken up by the iron/H^+^ cotransporter DMT1. In Caco-2 human intestinal epithelial cells, a consequence of ROS production due to overdoses of iron is a reduced plasma membrane level of DMT1, possibly as a consequence of its iron-induced endocytosis, thereby decreasing iron uptake rates [101].

In contrast, iron deficiency leads to adaptive responses that involve an increase in the activity of iron-transporters in the small intestine as well as morphological adaptations in the intestinal mucosa such as an increase in its thickness and the length of the villi to enhance the absorption of iron by enterocytes [102]. The surface expression of transferrin receptor is also maximized in many other cell types [100].

#### Platinum

Platinum drugs such as Cisplatin are common standard treatments for many types of tumors. One of their side effects is nephrotoxicity. A possible mechanism underlying Cisplatin-induced proteinuria is the decreased CME of proteins in the proximal tubule and the inhibition of the vATPase [103]. Cisplatin has also been proposed to stimulate EGFR endocytosis. However, in this case EGFR does not appear to be degraded in lysosomes or recycled to the plasma membrane, but accumulates in a subpopulation of non-degradative perinuclear multivesicular bodies. In contrast to normal EGFR internalization, the Cisplatin-dependent EGFR endocytosis does not require ubiquitination, but is Dynamin- and AP-2 dependent. This pathway appears to be crucial for survival as AP-2 loss induces apoptosis of Cisplatin-treated cells [104].

#### Heavy metals

Prolonged exposure to heavy metals such as cadmium, arsenic, mercury and lead is known to induce deleterious health effects, since they can bind to proteins and replace the original metals causing protein malfunctioning. At the cellular level, several studies have indicated oxidative stress leading to apoptosis as a common mechanism underlying their toxicity [105]. As metal ions can enter into the cell via endocytosis complexed to chelating proteins such as metallothioneins, it is easy to conceive that decreasing endocytic rates can be considered as a protective mechanism to avoid metal-induced toxicity. In agreement, several studies have reported a downregulation of endocytosis in proximal tubule cells exposed to cadmium [106] and a reduced endocytosis/phagocytosis capacity in dendritic cells and macrophages subjected to arsenic stress [107–109]. However, downregulation of endocytosis to bypass metallotoxicity has harmful side effects such as cadmium-induced proteinuria or arsenic-induced immunosuppression.

### Radiation stress

Radiotherapy is a common cancer treatment due to its contribution to cell death. It is known that penetrating radiation leads to cellular stress [110], mainly via ROS generation, protein misfolding, DNA breaks and organelle damage. However, radiotherapy has limitations due to cytotoxic effects on healthy cells and the associated radioresistance. Several papers have shown a differential impact of radiation on endocytosis. For example, defects in the migratory capacity of Langerhans cell-like dendritic cells subjected to ultraviolet B radiation was related to reduced receptor-mediated and fluid-phase endocytosis [111]. In contrast, work by [112] suggests that albumin uptake and fluid-phase endocytosis remain normal in irradiated inmature and mature dendritic cells. Carbon ion irradiation is also used as cancer therapy, being considered more effective than photon irradiation. This type of radiation was shown to promote apoptosis in both immature and mature dendritic cells, in part due to impaired endocytosis [113]. Like other types of stress, e.g. cisplatin, irradiation also promotes ligand-independent endocytosis of EGFR [114, 115] via an unknown mechanism.

## OUTLOOK

Beyond the examples provided here, there is little systematic knowledge about (i) which specific alterations in endocytosis are triggered by different stress conditions, (ii) how stress triggers changes in the overall endocytic capacity, (iii) which changes in the endocytic sorting of individual proteins are induced by stress, and (iv) how those changes contribute to a successful cellular adaptation to stress. Clearly, more systematic and unbiased approaches are needed to compare the effects of different stressors on endocytic flux in general and on specific endocytic cargo proteins.

In addition, it is increasingly being recognized that stress responses are not confined to individual cells. To coordinate adaptive responses within the organism, stressed cells communicate their status to their environment, either by displaying specific proteins on their surface or by releasing signal factors [116], e.g. with the help of surface-localized transport proteins. These aspects of the stress response could be supported by alterations in endocytosis. However, so far this possibility has not been investigated.

Our lack of understanding of the adaptation of endocytosis under stress conditions goes hand in hand with our limited knowledge about endocytic regulation in other physiological conditions. Even though numerous kinase pathways have been implicated in the regulation of endocytosis [27, 117] including some recent additions like the NIMA family kinases in *C. elegans* [118], how different signal transduction cascades modulate specific endocytic factors and cargo sorting in most cases has remained enigmatic. In addition to phosphorylation, ubiquitination events play a critical role in regulating the uptake of specific cargo proteins. Moreover, recent data suggest that both endocytosed cargo proteins and endocytic factors such as AP180 and AAK1 (reviewed in [119]) can also be subject to modification by carbohydrates such as O-GlcNAc that may regulate endocytosis by so far unknown mechanisms. Aside from these post-translational mechanisms, the regulation of endocytosis at the transcriptional level including the possible role of alternative splicing is only now starting to be appreciated. With advanced mass spectrometry and RNAseq-based approaches that allow to study different posttranslational modifications as well as mRNA abundance and splicing, it should become feasible to map the transcriptional and posttranslational regulation of cargo proteins and their associated endocytic factors upon different stress conditions and to delineate the underlying signaling pathways. This will pave the way for a more detailed understanding of how endocytosis is employed by mammalian cells to promote survival under stress.

## Abbreviatons:

AMPK: – AMP-activated kinase,
Bsep: – bile salt export pump,
CME: – Clathrin-mediated endocytosis,
EGFR: – epidermal growth factor receptor,
ENaC: – epithelial sodium channel,
GDI: – guanyl-nucleotide dissociation inhibitor,
GPCR: – G-protein coupled receptor,
HIF: – Hypoxia inducible factor,
LDL: – low density lipoprotein,
LDLR: – LDL receptor,
OGD: – oxygen and glucose deprivation,
ROS: – reactive oxygen species.
